# Fabrication of a 3D Printed Continuous Carbon Fiber Composite Grid Stiffened Structure Using Induction Heating

**DOI:** 10.3390/polym15183743

**Published:** 2023-09-13

**Authors:** Zhuoying Zhou, Zhongsen Zhang, Kunkun Fu, Bin Yang

**Affiliations:** School of Aerospace Engineering and Applied Mechanics, Tongji University, Shanghai 200092, China

**Keywords:** 3D printing, induction heating, continuous fiber reinforced composite grid, mechanical properties

## Abstract

In aerospace applications, composite grids have been widely utilized to enhance the strength of large thin-shell components. Recently, a growing focus has been on the research of 3D printing continuous fiber-reinforced thermoplastic composites. The 3D printing method offers various advantages over traditional molding processes, including a simpler process, higher material utilization, and lower manufacturing costs. However, the use of 3D printing for manufacturing continuous fiber-reinforced composite structures presents challenges, such as a high occurrence of defects within the structure and insufficient mechanical properties. These limitations hinder its widespread application. To address these issues, this study proposes a method for treating 3D-printed composite grid structures using induction heating. Initially, the induction heating mechanism of 3D-printed composite grids was analyzed by studying the impedance at the junction, including direct contact resistance and dielectric hysteresis loss. Subsequently, the impact of induction heating treatment on internal defects was explored by observing micro morphologies. The results show that the combination of induction heating and vacuum pressure effectively reduces porosities within the 3D-printed carbon fiber composite grids. Additionally, 3D-printed composite grid-stiffened PLA structures were fabricated with induction heating, and the bending and impact tests were conducted to evaluate their mechanical properties. The results indicate that using a grid-unit size of 4 mm leads to significant increases in bending strength and modulus of the grid-stiffened structure, with improvements of 137.6% and 217.8%, respectively, compared to the neat PLA panel. This demonstrates the exceptional mechanical enhancement efficiency of the 3D-printed lightweight composite grids.

## 1. Introduction

In recent years, high-performance fiber-reinforced composites have received increasing attention as an effective alternative material to metal grid structures [1,2] due to their lightweight, easy processing, recyclability, and high-specific mechanical properties [3,4]. Among the main load-bearing structures of spacecraft, grid structures made from lightweight high-performance fiber-reinforced composites have been rapidly developed [5]. Specifically, carbon fiber composite structures with low coefficients of thermal expansion are of particular interest due to their potential applications in satellite frame structures that require high dimensional stability [6].

Compared to traditional composite molding processes, 3D printing methods offer several advantages [7]. These include the ability to produce structures with high dimensional accuracy, efficient material utilization, and the capacity for integrated and rapid formation of complex structures [8]. As a result, 3D printing technology is well suited for fabricating complex forms of grid structures using continuous fiber-reinforced thermoplastic composites [9,10].

The 3D printing of continuous fiber-reinforced thermoplastic composite structures exhibits numerous defects and insufficient mechanical properties, which restricts its broad application [11]. In comparison to composite grid structures produced through traditional process methods, the 3D printing technique is a short-time low-pressure molding process, resulting in a high internal porosity [12]. However, porosity serves as a critical factor influencing the mechanical properties of composite structures [13]. Consequently, a method for treating high-porosity 3D-printed continuous fiber-reinforced thermoplastic composite grid structures needs to be proposed to mitigate the defects. One effective approach to improve the mechanical performance of 3D-printed continuous fiber-reinforced thermoplastic composite structures is by utilizing post-processing techniques involving hot pressing [12,14]. Ovens and autoclaves are commonly employed as heat sources. Induction heating technology employs an alternating magnetic field to induce eddy currents in a conductive circuit, thereby generating Joule heat. This non-contact heating technique allows for localized heating without the need for direct contact with the heated object [15,16]. In comparison to traditional heating methods, induction heating presents several advantages, including high heating efficiency, low energy consumption, non-contact operation, and localized heating capability [17,18]. With the development of carbon fiber-reinforced thermoplastic composite materials, induction heating technology has also been used for induction heating technology for the welding of thermoplastic components and metal/thermoplastic hybrid materials [19]. This technology provides a localized, non-contact heat source that is suitable for forming, connecting, and assembling carbon fiber/thermoplastic composite components [20]. The periodic conductive circuit of continuous carbon fiber-reinforced composite grid structures makes them ideal for induction heating [21]. Inducing heating generates the Joule heating effect in the conductive circuit of the grid structure, resulting in localized heat generation [15,22,23]. Applying pressure while utilizing induction heating has the potential to significantly diminish the porosity in composites. This makes induction heating a highly promising post-processing technique for 3D-printed carbon fiber composite grid structures.

In this study, a method utilizing induction heating for treating the 3D-printed continuous carbon fiber-reinforced composite grid structures was proposed. The induction heating mechanism of 3D-printed composite grids was initially analyzed by investigating the impedance at the junction using an electrothermal model and infrared thermal imaging. The impact of induction heating treatment on internal defects was then explored by observing micro morphologies. Finally, 3D-printed composite grid-stiffened PLA structures were fabricated with induction heating, and the bending and impact tests were conducted to evaluate their mechanical properties.

## 2. Materials and Methods

### 2.1. Materials

Lab-made carbon fiber (CF) reinforced polylactic acid (PLA) prepreg filaments were employed to print composite grids. The prepreg filaments with a diameter of 0.5 mm were prepared by impregnating a carbon fiber bundle (T300-1K, Toray, Tokyo, Japan) with PLA resin (4032D, NatureWorks, Minneapolis, MN, USA), as described in our previous work [24]. PLA 3D-printed filaments with a diameter of 1.75 mm, purchased from Polymaker Inc., Changshu, China, were used for the preparation of the thermoplastic panel.

### 2.2. 3D Printing of CF/PLA Composite Grid Stiffened Structures

**Figure 1:**
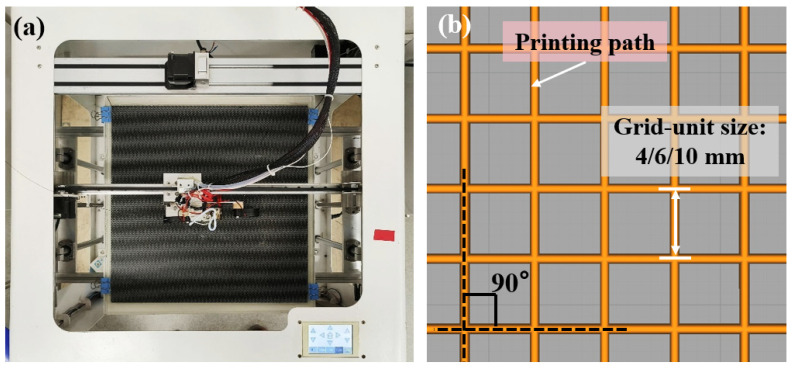
(**a**) Photograph of the customized 3D printing system and (**b**) schematic drawing of the 3D-printed composite grids.

A customized 3D printing system is employed to fabricate composite grid structures, as shown in Figure 1a. A continuous fiber 3D printer was custom based on a commercial FDM device (Jiuyuezhuofei Co., Ltd., Beijing, China). Specifically, the feed inlet was firstly optimized to ensure compatibility with continuous prepreg filaments. Additionally, a custom 1.2 mm diameter nozzle with a flat bottom was utilized to effectively flatten the circular prepreg filament into a strip [25]. The detailed processing parameters are listed in Table 1. As illustrated in Figure 1b, square grids with different unit sizes (4, 6, 10 mm) were prepared by using the continuous CF/PLA prepreg filaments.

Rectangular PLA plates with a thickness of 2 mm were individually fabricated using the Infinity X1, a commercial FDM 3D printer. These plates served as panels for the grid-stiffened structures.

### 2.3. Induction Heating Processing of CF/PLA Composite Grid Stiffened Structures

**Figure 2:**
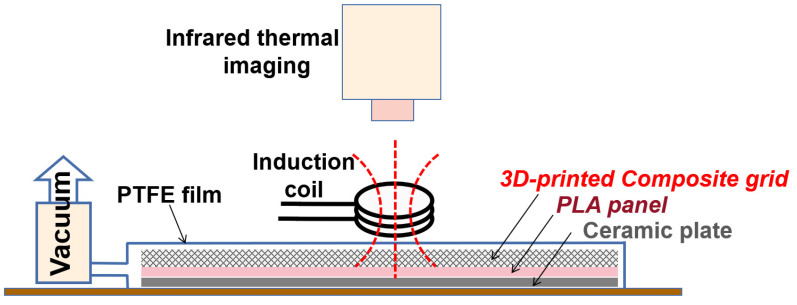
Schematic diagram of induction heating process.

An electromagnetic induction heating device was utilized to process the 3D-printed composite grid. The device operated at a frequency of 800 kHz and had a maximum power output of 5.0 kW. An induction coil with an internal diameter of 8 mm was employed to generate the high-frequency oscillating magnetic field. As depicted in Figure 2, the induction coil was positioned parallel to the grid specimen, maintaining a distance of 5 mm. Throughout the induction heating process, a vacuum pressure was applied to the composite grids using a thin PTFE vacuum bag, and a ceramic plate was employed to preserve their shape. The composite grid was moved across the induction coil plane to ensure that every part of the grid was heated up to the melting point of the PLA resin, given the limited effective induction heating area. The heating process was monitored using a thermal infrared imaging camera. The vacuum pressure was maintained until the composite grid completely cooled down in order to reduce any potential warping of the structure.

### 2.4. Induction Heating Mechanism Analysis

**Figure 3:**
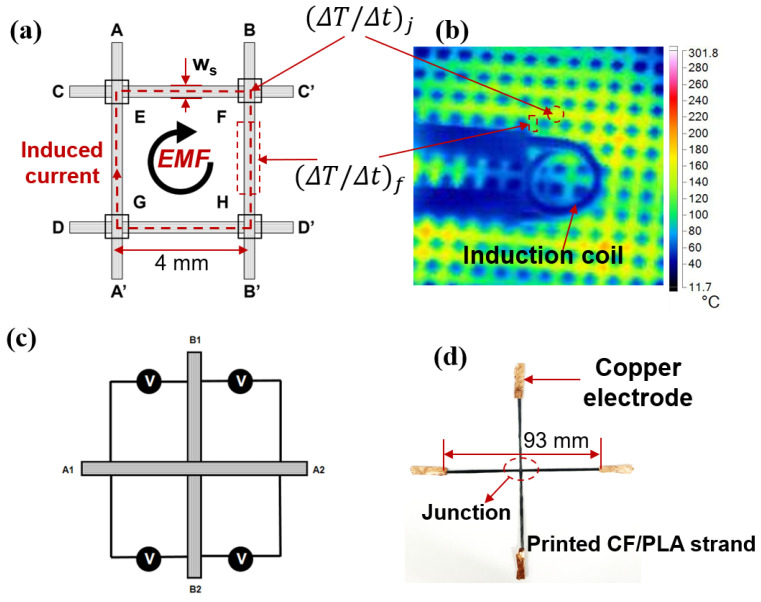
Schematic diagram of resistance measurements at junctions: (**a**,**b**) impedance at high-frequency induction current, and (**c**,**d**) direct contact resistance.

When subjected to a high-frequency electromagnetic field, the continuous carbon fiber reinforced composite grid structure will generate an induced current as a result of the induced electromotive force (EMF) due to the conductivity of carbon fiber filaments, as depicted in Figure 3a. The induced current circuit within the carbon fiber composite grid structure generates both Joule heat and dielectric heating, eventually causing the thermoplastic resin to melt. The induction heating region of the composite grid structure can be divided into two parts: four carbon fiber strands and four junctions [26]. The induction heating mechanisms of composite grid structures can be categorized as follows: Joule heating of carbon fiber strands, dielectric hysteresis loss heating at the junction, and Joule heat caused by fiber contact resistance at the junction.

In the case of Joule heating of carbon fiber strands, the induced EMF forms a conductive circuit within the carbon fiber strands, resulting in the generation of Joule heat due to the inherent resistance of carbon fibers [21]. Dielectric hysteresis loss heating at the junction is the main mechanism of heating for matrix materials with electrically insulating properties [22]. When there is no direct contact between the two bundles of carbon fibers at the junction, i.e., the fibers are separated by the wrapped insulating resin, the resistance at the junction is considered to be equivalent to a parallel capacitance. Heat is then produced through dielectric hysteresis loss. In the case of Joule heat caused by fiber contact resistance at the junction, when direct contact occurs between the fibers at the junction, forming a closed circuit, an electric current is generated, resulting in heating [27].

The above is the basic mechanism of induction heating for each unit of the carbon fiber-reinforced composite grid structure. In this case, the resistance of the fiber bundle can be obtained by direct measurement. However, it is more difficult to evaluate the resistance at the junctions to determine whether it is dielectric hysteresis loss heating or contact Joule heat heating mechanism. Therefore, in this paper, the induction heating mechanism of the grid structure is elucidated by measuring the resistance at the junction of the induction heating process as described below [28].

To evaluate the induction heating mechanism of the 3D-printed composite grids, an electrical model was established, as shown in Figure 3. The ratio of the heat generated in each carbon fiber strand ($P_{f}$) and junction ($P_{j}$) can be calculated by the following Equation (1),

(1) $$\frac{P_{j}}{P_{f}}=\frac{R_{j}}{R_{f}}=\frac{\Lambda }{\rho_{f}}\left(\frac{v_{f}w_{s}t_{s}}{l}\right)$$

$R_{f}$ is the resistance of the carbon fiber strand. $R_{j}$ is the resistance at the junction, which is denoted as Λ at high-frequency-induced current. $\rho_{f}$ is the resistance of carbon fibers. $v_{f}$ is the fiber volume fraction of CF/PLA strands. $w_{s}$ and $t_{s}$ are the width and thickness of the 3D-printed CF/PLA strand, respectively, and l is the grid-unit size.

The volume occupied by carbon fiber strands ($V_{f}$) and junction ($V_{j}$) is expressed as:

(2) $$V_{f}=v_{f}\left(w_{s}\cdot t_{s}\cdot l\right)$$

(3) $$V_{j}=w_{s}\cdot w_{s}\cdot 2t_{s}$$

The ratio of the heating intensity at the junction and at the fiber strands can be expressed as:

(4) $$\frac{P_{j}{}^{\prime \prime \prime }}{P_{f}{}^{\prime \prime \prime }}=\frac{P_{j}/V_{j}}{P_{f}/V_{f}}=\frac{\Lambda v_{f}{}^{2}t_{s}}{2\rho_{f}}$$

$P_{j}{}^{\prime \prime \prime }$ are $P_{f}{}^{\prime \prime \prime }$ are the heating rate per unit volume in the fibers of each conductive strand and at each junction of the grids. The heat loss generated is assumed to be negligible in a short period of time. The volumetric heat capacity ($\rho C_{p}$) is assumed to be constant so that the rate of temperature change at each fiber or junction can be approximated by the following Equation:

(5) $$\frac{\Delta T}{\Delta t}\approx \frac{P^{\prime \prime \prime }}{\rho C_{p}}$$

The resistance at the junction can be estimated from the measured temperature change rate for each grid cell by the following Equation, as follows:

(6) $$\Lambda \approx \left(\frac{2\rho_{f}}{v_{f}{}^{2}t_{s}}\right)\frac{{\left(\Delta T/\Delta t\right)}_{j}}{{\left(\Delta T/\Delta t\right)}_{f}}$$

A CF/PLA composite grid, with a grid-unit size of 4 mm, was fabricated and treated using induction heating in this study. Using infrared thermal imaging, the rate of temperature change at the junctions (${\left(\Delta T/\Delta t\right)}_{j}$) and at the fiber strands (${\left(\Delta T/\Delta t\right)}_{f}$) are recorded and then measured to estimate the Λ, as shown in Figure 3b. To distinguish the heating mechanism at the junction, which might include dielectric hysteresis loss and Joule heating of direct contact resistance ($R_{jd}$), the $R_{jd}$ was further measured using a two-probe method. As illustrated in Figure 3c,d, two cross CF/PLA strands were printed on the bed using the same processing parameters listed in Table 1. The ends of the printed CF/PLA strands were heated at 400 °C for 10 s to remove the wrapped PLA resin and then mounted with copper electrodes, reducing the impact of contact resistance between the strand ends and the measuring probe. All the resistances at each two ends (e.g., A1A2, B1B2, A1B1, A2B1) were measured and recorded. The $R_{jd}$ can be estimated by calculating the difference in resistance values between each two ends.

### 2.5. Mechanical Characterization of 3D-Printed Composite Grid Stiffened Structures

**Figure 4:**
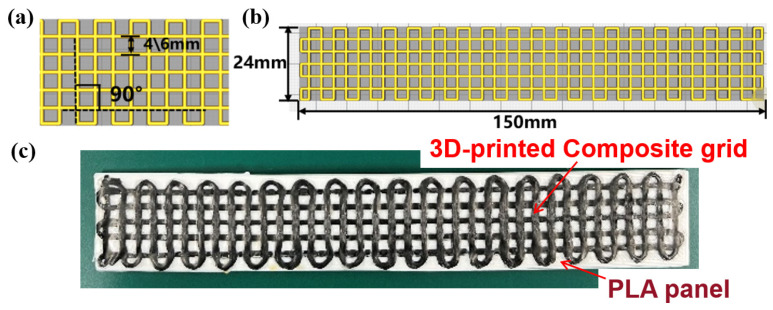
(**a**,**b**) Dimensional parameters and (**c**) photograph of CF/PLA composite grid stiffened PLA specimens for bending tests.

According to the processing parameters listed in Table 1, continuous CF/PLA grids with varying grid-unit sizes of 4mm and 6 mm were prepared and denoted as CF-B-04 and CF-B-06, respectively. The CF/PLA grid stiffened PLA structure was obtained by induction heating treatment and vacuum pressure, as depicted in Figure 4. The bending properties of the 3D-printed CF/PLA grid stiffened PLA structures were tested using a universal material testing machine with a span of 64 mm and a loading rate of 2.0 mm/min, according to ASTM D7264 standard [29]. Each group was tested with a minimum of three specimens.

**Figure 5:**
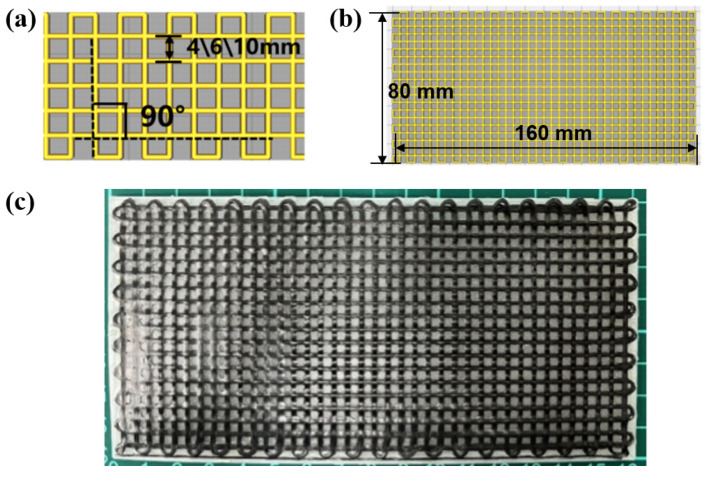
(**a**,**b**) Dimensional parameters and (**c**) photograph of CF/PLA composite grid stiffened PLA specimens for low-velocity impact tests.

To investigate the low-velocity impact properties of the composite grid structures, specimens with varying grid-unit sizes of 4 mm, 6 mm, and 10 mm were prepared and denoted as CF-I-04, CF-I-06, and CF-I-10, respectively, as illustrated in Figure 5. The processing parameters for impact test specimens are the same as those for bending test. The impact tests were conducted using an Instron drop-weight impact testing machine according to ASTM D7136 standard [30]. The mass and diameter of the impactor are 2.254 kg and 16.5 mm, respectively. The impactor was set to an initial velocity of 1.52 m/s, with an impact energy of 2.8 J. Each group was tested with a minimum of three specimens.

### 2.6. Morphology Characterization

The surface morphology, microstructure, and defects of 3D-printed CF/PLA composite structure before and after induction heating treatment were observed and analyzed by SEM (Sigma 300, ZEISS, Oberkochen, Germany) and Micro-CT (NanoVoxel-3000, SANYING, Tianjin, China).

## 3. Results and Discussion

### 3.1. Induction Heating Mechanisms of CF/PLA Grid Structures

**Figure 6:**
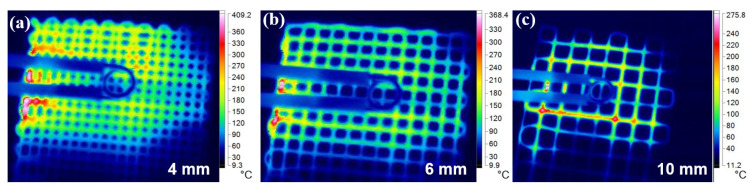
Infrared thermal images of induction-heated CF/PLA grids with different grid-unit sizes: (**a**) 4 mm, (**b**) 6 mm, and (**c**) 10 mm.

Figure 6 depicts the temperature distribution of the 3D-printed CF/PLA grids with varying grid-unit sizes subjected to a 12-s induction heating process. The temperature distribution is highly uniform for the 4 mm and 6 mm grids, but there is a noticeable heat concentration at the junctions. This phenomenon can be explained by the induction heating mechanism of the composite grids, which will be further discussed. Additionally, it is observed that the effective heating zone for all grids is approximately 40 mm × 40 mm. The size and distance of the induction coil primarily influence this area. In this study, the use of an 8 mm induction coil resulted in an effective heating area that is 25 times larger than its own area, which greatly improves the processing efficiency of the induction heating treatment.

**Figure 7:**
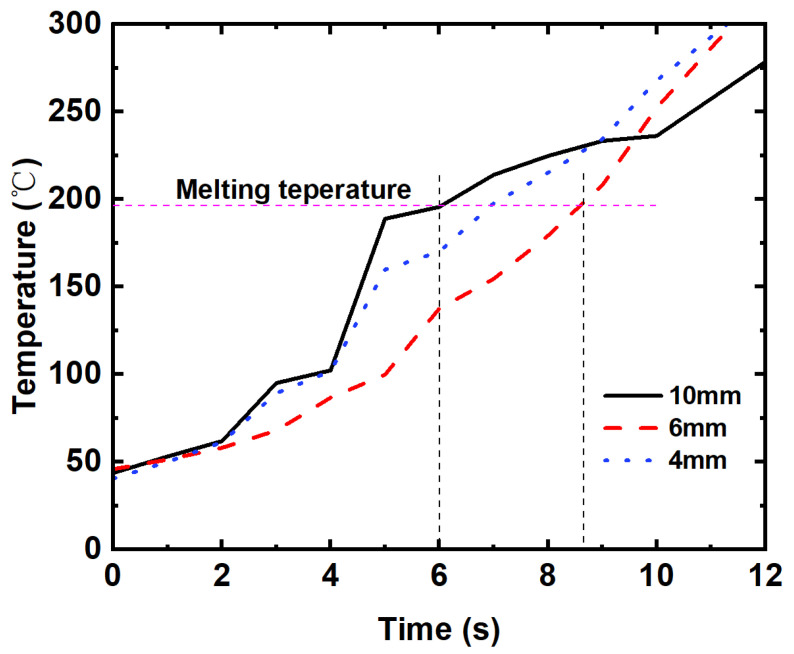
Heating curve of 3D-printed CF/PLA grids with different grid-unit sizes: (blue line) 4 mm, (red line) 6 mm, and (black line) 10 mm. (The black dashed lines indicate the moment at which the temperature reaches the melting point).

The heating curves for these three grids during the induction heat processing are presented in Figure 7. The results demonstrate the significantly high heating efficiency of induction heating and the insensitivity of the heating rate to the grid-unit size. All the grids could be heated above their respective melting points within 9 s, showcasing a much higher speed compared to conventional heating methods such as oven heating.

Table 2 presents the measured resistance of 2 printed CF/PLA strands using the 2-probe method. From the table, it is evident that the resistance between the ends of A1B1, A1B2, A2B1, and A2B2 is only slightly higher than that between A1A2 and B1B1. This indicates that there was a direct contact between the two cross-printed strands, resulting in an average contact resistance of *R_jd_* 16.51 Ω for an overlap area of 1.5 mm × 1.5 mm. Considering the relatively low resistance of printed carbon fiber strands (0.4 Ω/mm), the larger *R_jd_* could lead to a twenty times more increase in the heat generated at the junction compared to the fiber strands. However, the findings do not align with the results observed in the infrared thermal images (Figure 6), which do not present noticeable heat concentration at the junctions. It demonstrates that the junction impedance at high-frequency-induced current is much lower than that at direct current (DC).

To further investigate the induction heating mechanism of the continuous fiber composite grid structure, the temperature distribution in the composite grid is monitored using an infrared thermal imager during the induction heating process. The grid-unit size of the composite grid used in this study is 4 mm. The temperature change rate at the fiber strands and junctions within a 3-s interval is recorded and presented in Table 3. In this research, the resistivity ($\rho_{f}$) of carbon fiber is determined to be 1.108 × 10^−5^ Ω∙m, while the thickness of the printed strand ($t_{s}$) is 0.2 mm, and the volumetric fraction of the fiber ($v_{f}$) is 30%. Based on Equation (6), the junction resistance (*Λ*) for high-frequency induction current is estimated to be 1.6 Ω. Notably, the junction resistance (*Λ*) at a current frequency of 800 K is significantly lower than the contact resistance (*R_jd_*) measured at DC. This observation suggests that the dielectric hysteresis loss plays a crucial role as a heating mechanism at the junction.

The lower resistance at the junction is advantageous in mitigating the temperature gradient difference between the fiber strands and the junctions. Nevertheless, the *Λ* value is still approximately three times higher than the resistance of carbon fiber strands. Consequently, the heat generated at the junctions is expected to be three times greater than that at the fiber strands. Analysis of infrared thermal images (Figure 6) reveals that the temperature gradient difference between the fiber strands and the junctions is insignificant for grids with small grid-unit dimensions (4, 6 mm). However, for grids with a grid-unit size of 10 mm, there is a noticeable concentration of heat at the junctions. This can be ascribed to the high thermal conductivity of carbon fibers, which facilitates the rapid transfer of heat from the junctions to the strands, resulting in a relatively uniform temperature distribution. When the grid-unit size becomes too large (e.g., 10 mm), the rate of thermal conduction becomes insufficient to maintain the temperature uniformity of the grid structure. Thus, it is crucial to carefully select an appropriate grid-unit size when designing composite grid structures for a specific frequency EMF.

### 3.2. Influence of Induction Heating Treatment on Internal Defects

**Figure 8:**
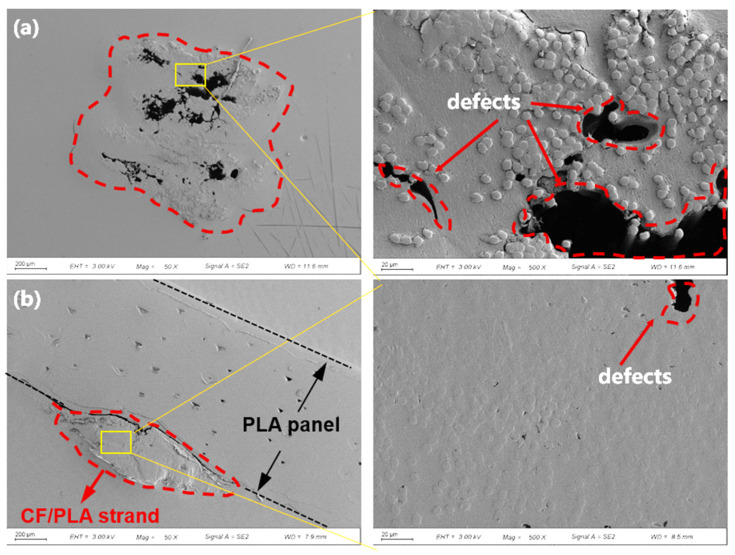
Cross-sectional morphologies of 3D-printed CF/PLA grid (**a**) before and (**b**) after induction heating.

The cross-sectional SEM image of the 3D-printed carbon fiber composite grid is shown in Figure 8a. It reveals that the circular prepreg filaments were ironed to flat strands by the nozzle during the 3D printing process. Importantly, there are multiple gaps or porosities present both between each printed layer and within the fiber strands, which is mainly ascribed to the relatively low printing pressure [31]. Figure 8b displays the cross-sectional morphologies of the 3D-printed carbon fiber composite grid after induction heating treatment. It is evident that the grid’s thickness has been decreased from 1 mm to approx. 0.2 mm, indicating some extent of flattening due to external vacuum pressure during the induction heating process. Moreover, the gaps between layers that existed prior to induction heating have significantly reduced. The porosities within the internal filament bundles have also diminished. Following induction heating, the resin fills the voids between the fibers, causing the fibers to be closer together. Some fibers have also become compressed or squashed. It demonstrates that the utilization of both induction heating and vacuum pressure as a post-treatment method effectively reduces the internal defects within the 3D-printed carbon fiber composite grids.

**Figure 9:**
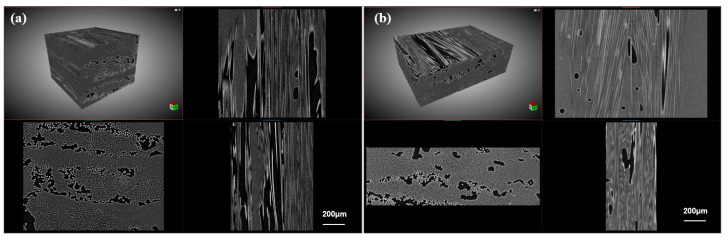
Micro-CT scan images of 3D-printed CF/PLA grid (**a**) before and (**b**) after induction heating.

The porosity of the 3D-printed CF/PLA grid before and after induction heating was further quantified using micro-CT scanning, as shown in Figure 9. The pristine porosity of the composite grid was 17.1%, which decreased to 8.7% after induction heating. It can be concluded that the instantaneous heat generated by the carbon fibers through induced current resulted in the surrounding PLA matrix melting rapidly. The melted PLA resin could re-impregnate the carbon fiber filaments under capillary action. Furthermore, it should be noted that induction heating is an internal heating method, which is entirely distinct from external heating methods like oven heating. The heating of carbon fiber itself creates a temperature gradient from the inside to the outside. As the PLA resin gets closer to the carbon fiber, it becomes hotter, resulting in a decrease in viscosity. This heat transfer manner facilitates the capillary diffusion of PLA resin into the voids within the bundles of carbon fibers. Additionally, the application of additional vacuum pressure accelerates the impregnation and fusion of the melted resin, leading to a considerable decrease in porosity.

### 3.3. Mechanical Performance of the 3D-Printed CF/PLA Grid Stiffened Structures

**Figure 10:**
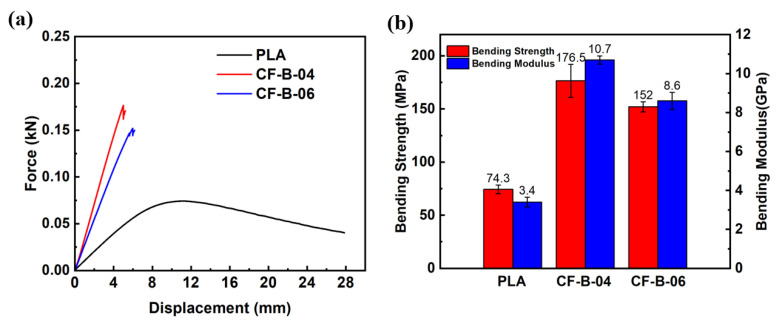
(**a**) Force-displacement curves and (**b**) calculated bending properties for 3D-printed PLA and CF/PLA grid stiffened structures.

The bending properties of 3D-printed PLA and CF/PLA grid-stiffened PLA structures are presented in Figure 10. It demonstrates that the bending properties of the PLA were significantly enhanced by stiffening with the 3D-printed CF-reinforced composite grids. Specifically, when the grid-unit size is 4 mm, the mass of the structure increases by 21.15% compared to the neat PLA panel, as listed in Table 4. However, the bending strength and modulus show remarkable increases of 137.6% and 217.8%, respectively, at fiber content of 5.2 wt.%. On the other hand, for the composite grid-stiffened structures with a grid-unit size of 6 mm, the fiber content decreases to 3.2 wt.%, but their specific bending strength and modulus still experience improvements of 82.9% and 133.0%, respectively. These findings highlight the exceptional mechanical enhancement efficiency of the 3D-printed lightweight composite grids.

**Figure 11:**
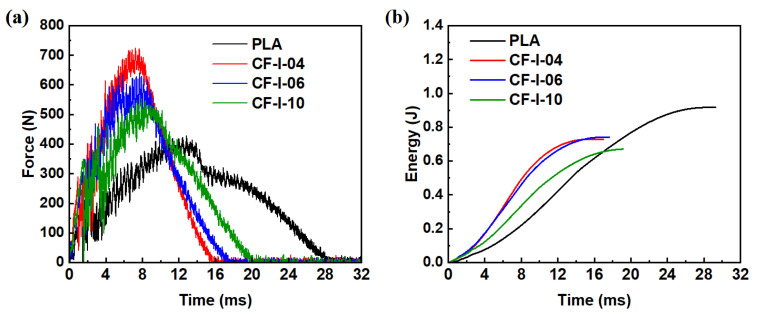
(**a**) Force and (**b**) energy versus time response of 3D-printed PLA and CF/PLA grid stiffened structures during the low-velocity impact tests.

Figure 11 illustrates the low-velocity impact response of 3D-printed PLA and CF/PLA grid-stiffened structures. The force-time curves clearly show that all the CF/PLA grid-stiffened structures have significantly higher stiffness compared to the neat PLA plate. As listed in Table 5, the peak loads for the grid-stiffened structures with grid-unit sizes of 4 mm, 6 mm, and 10 mm were increased by 69.4%, 49.7%, and 27.9%, respectively, demonstrating a significant improvement in impact resistance.

During an impact event, energy is absorbed by a material through elastic and plastic deformation, as well as the occurrence of failures. Neat PLA structures can absorb impact energy through a combination of elastic and plastic deformation. However, due to the high stiffness of grid-stiffened PLA structures, the impact energy was absorbed mainly through elastic deformation, resulting in a 14–27% reduction in absorbed energy for different grid-stiffened structures.

## 4. Conclusions

In this study, a novel treatment method for 3D-printed composite grid structures was proposed using induction heating. The induction heating mechanism of 3D-printed composite grids was first analyzed by studying the impedance at the junction, including direct contact resistance and dielectric hysteresis loss. Subsequently, the impact of induction heating treatment on internal defects was explored by observing micro morphologies. Additionally, 3D-printed composite grid-stiffened PLA structures were fabricated with induction heating, and the bending and impact tests were conducted to evaluate their mechanical properties. The following conclusions could be drawn as follows:

(1) The infrared thermal imaging demonstrates the significantly high heating efficiency of induction heating and the insensitivity of the heating rate to the grid-unit size. The grids could be rapidly heated above their respective melting temperatures within 9 s. However, when the grid-unit size becomes too large, it would lead to a non-uniform temperature distribution within the grid structure. Thus, it is crucial to carefully select an appropriate grid-unit size when designing composite grid structures for a specific frequency EMF.
(2) The observation of microstructures shows that the combination of induction heating and vacuum pressure effectively reduces porosities within the 3D-printed carbon fiber composite grids due to the special internal heating mechanism.
(3) The bending test results indicate that using a grid-unit size of 4 mm leads to significant increases in bending strength and modulus of the grid-stiffened structure, with improvements of 137.6% and 217.8%, respectively, compared to the neat PLA panel. This demonstrates the exceptional mechanical enhancement efficiency of the 3D-printed lightweight composite grids.
(4) A significant improvement in impact resistance was observed for the PLA panels stiffened with CF/PLA grids. The peak loads for the grid-stiffened structures with grid-unit sizes of 4 mm, 6 mm, and 10 mm were increased by 69.4%, 49.7%, and 27.9%, respectively.

The 3D-printed lightweight grid-stiffened thin-walled thermoplastic structures have potential applications in civil aircraft for non-load-bearing functional structures, including cabinets, protective covers, maintenance covers, and more.

## Figures and Tables

**Table 1:** 3D printing parameters for composite grids.

Printing Parameters	Values
Layer thickness	0.25 mm
Nozzle temperature	205 °C
Printing speed	7.5 mm/s
Grid-unit size	4/6/10 mm
Grid thickness	1 mm

**Table 2:** Junction contact resistance measurement results of 3D-printed CF/PLA grids.

	A1A2	B1B2	A1B1	A1B2	A2B1	A2B2
Resistance (Ω)	37.98	36.57	54.52	54.59	52.70	53.34
Direct contact resistance at the junction (*R_jd_*) (Ω)	/		17.24	17.31	15.42	16.06
			average: 16.51			

**Table 3:** Temperature change rate at fiber strands and junctions during induction heating process.

	AA’	BB’	CC’	DD’	E	F	G	H	Average Value
${\left(\Delta T/\Delta t\right)}_{f}$ (°C/s)	6.2	5.5	5.5	5.2	/				5.6
${\left(\Delta T/\Delta t\right)}_{j}$ (°C/s)	/				7.0	7.3	7.7	7.1	7.1
$\frac{{\left(\Delta T/\Delta t\right)}_{j}}{{\left(\Delta T/\Delta t\right)}_{f}}$	1.3								

**Table 4:** Physical and bending properties of 3D-printed PLA and CF/PLA grid stiffened structures.

Samples	Mass (g)	Carbon Fiber Content (wt%)	Bending Strength (MPa)	Bending Modulus (GPa)	Specific Bending Strength (MPa/g)	Specific Bending Modulus (GPa/kg)
CF-B-04	11.14	5.2	176.5 ± 15.5	10.74 ± 0.21	15.84 ± 0.24	964.05 ± 38.20
CF-B-06	10.30	3.2	152.0 ± 4.8	8.60 ± 0.44	14.76 ± 0.14	855.35 ± 33.07
PLA	9.21	0	74.3 ± 4.10	3.38 ± 0.25	8.07 ± 0.46	367.10 ± 42.75

**Table 5:** Impact properties of 3D-printed PLA and CF/PLA grid-stiffened structures.

Samples	Mass (g)	Carbon Fiber Content (wt%)	Maximum Energy (J)	Peak Load (N)
CF-I-04	39.31	5.7	0.79 ± 0.05	724.81 ± 44.17
CF-I-06	37.04	4.2	0.74 ± 0.03	640.40 ± 78.59
CF-I-10	35.25	2.9	0.67 ± 0.18	547.25 ± 4.36
PLA	31.87	0	0.92 ± 0.23	427.90 ± 4.95

## Data Availability

Data available on request due to restrictions of privacy.
